# Long-term infection risks in haematological cancer survivors compared with individuals with no cancer history: protocol for a systematic review aided by artificial intelligence-based methods

**DOI:** 10.1136/bmjopen-2025-114803

**Published:** 2026-03-18

**Authors:** William Wilson, Harriet Forbes, Matthew Hazell, Lily Hopkins, Garth Funston, Maeve O’Reilly, Krishnan Bhaskaran, Helena Carreira

**Affiliations:** 1 Department of Non-Communicable Diseases Epidemiology, London School of Hygiene & Tropical Medicine, London, UK; 2 Wolfson Institute of Population Health, Queen Mary University of London, London, UK; 3 University College London Hospitals NHS Foundation Trust, London, UK; 4 University College London Cancer Institute, London, UK

**Keywords:** Leukaemia, Lymphoma, Myeloma, INFECTIOUS DISEASES, Systematic Review

## Abstract

**Abstract:**

**Introduction:**

Infections are a major cause of morbidity and mortality among individuals with haematological cancers, but the duration of elevated risk in long-term survivors remains uncertain. Although previous attempts to summarise the existing literature on this topic would have been hampered by the sheer volume of studies on cancer and all-cause infections, emerging artificial intelligence tools now offer the ability to streamline the screening process, allowing for broader and more comprehensive reviews.

**Methods and analysis:**

This protocol follows the Preferred Reporting Items for Systematic Reviews and Meta-Analysis Protocols guidelines. Eligible studies will include original observational data reporting long-term (≥1 year follow-up from diagnosis) infection-related outcomes in haematological cancer survivors compared with a general or cancer-free population. Screening will be supported by ASReview, an artificial intelligence-based tool for abstract prioritisation. An internal validation step will be conducted by comparing artificial intelligence-assisted screening results with manual review performed by two independent researchers on a subset of abstracts. The primary outcomes of infection incidence and infection mortality will be summarised by type of infection, type of haematological cancer and time since cancer diagnosis. Information on anti-cancer treatments received will also be described. Data synthesis will be mostly narrative due to the broad scope of the review, though meta-analyses will be performed in cases where studies are sufficiently homogenous. Risk of bias will be assessed using the Newcastle-Ottawa Scale.

**Ethics and dissemination:**

Ethical approval is not applicable to this study. The results of the review will be disseminated to clinical audiences and submitted to a peer-reviewed journal.

**PROSPERO registration number:**

CRD420251047091.

**STRENGTHS AND LIMITATIONS OF THIS STUDY**

- Innovative use of artificial intelligence methods with robust internal validation.
- Inclusion of all infection types and haematological malignancies provides a comprehensive summary of long-term infection risks across diverse survivor groups.
- Searches will be conducted in the two largest and most comprehensive biomedical literature databases (MEDLINE and Embase) with no restrictions on publication date or geography.
- Heterogeneity in study populations, outcomes and effect measures may limit comparability and the potential for quantitative synthesis.
- Restricting inclusion to English-language studies may lead to omission of relevant non-English publications.

## Background

 Haematological malignancies such as leukaemia, lymphoma and multiple myeloma, among others,^1^ represent a significant burden on public health. Globally, non-Hodgkin's lymphoma and leukaemia ranked as the 10th and 13th most common cancers in 2022,^2^ while among children aged 0–14 years, leukaemia is by far the most incident malignancy worldwide.^3^ Increasing cancer incidence^4^ and survival rates^4 5^ have led to an ever-growing population of cancer survivors. In the UK alone, the 5-year prevalence of leukaemia, lymphoma and multiple myeloma combined is estimated at 170 per 100 000 (~115 000 individuals).^6^ This large number of individuals living with and beyond the disease is susceptible to a variety of late effects of cancer and its treatments.^7^

Infections are a major cause of concern for patients with haematological cancer, both due to intrinsic disease-related immune defects and the immunosuppressive nature of the treatments.^8 9^ Data from clinical trials may give an accurate estimate of the risk of infection for patients on active anticancer treatment. However, the generalisability of these studies is often limited by highly selective eligibility criteria, and follow-up is rarely extensive enough to describe the enduring risk of infection following the completion of treatment, particularly in the long term.

Large cohort studies focusing on specific infections such as influenza^10^ and COVID-19^11^ have highlighted adverse clinical outcomes for real-world haematological cancer survivors at over 10 and 5 years from diagnosis, respectively. However, these studies are limited by their focus on individual infection types and do not provide insight into the full spectrum of infectious complications that survivors may experience over their lifetime. In addition, haematological cancers are often grouped together, overlooking potential differences in infection risk between subtypes. Other studies may instead focus on a single subtype, limiting opportunities for comparison across the full range of blood cancers. A systematic review that synthesises evidence across all infection types and haematological cancers would provide a more comprehensive picture of long-term infection risk and support more targeted survivorship care.

Until recently, it was not feasible to comprehensively characterise the body of literature on all-cause infections in haematological cancer survivors simply due to the sheer volume of research spanning these areas. However, recent developments in artificial intelligence-based natural language processing (NLP) algorithms offer an alternative to traditional literature screening methods and have been shown to effectively speed up the review process when used appropriately.^12 13^ The application of NLP algorithms is particularly well suited to this setting where, for example, search expressions may struggle to differentiate the large volume of research on infection as a risk factor for cancer and infection as an outcome in cancer groups. Similarly, search expressions that may filter out studies with no relevant comparison cohort (ie, the general population or those without cancer) are too restrictive and run the risk of missing relevant articles, potentially identifying a biased sample of studies.

This protocol describes the a priori defined methodology for a systematic review using an NLP algorithm-based tool to summarise the existing evidence on how infection risk in long-term haematological cancer survivors compares to those without cancer or the general population. The review will also examine how infection-related risks vary by type of cancer, cancer treatment and time since cancer diagnosis, with a particular focus on the long-term, survivorship period.

## Methods and analysis

This protocol follows the Preferred Reporting Items for Systematic Reviews and Meta-Analysis Protocols (PRISMA-P) guidelines.^14^ The PRISMA-P checklist is available in online supplemental file 1. The review has been registered with the International Prospective Register of Systematic Reviews (PROSPERO 2025 CRD420251047091) with a start date of 6 May 2025 and initial anticipated completion of 31 August 2025. Due to the volume of eligible studies, the anticipated completion date has been updated in PROSPERO to 30 April 2026.

### Eligibility criteria

To be eligible for inclusion in the review, studies must be based on original data, use a longitudinal observational study design and include infection-related outcome data (eg, incidence, hospitalisation, mortality) on both a cohort of haematological cancer survivors and a comparison cohort of either individuals with no history of cancer or the general population. Studies that describe outcomes for a specific infection as well as those that investigate infection as a broad category will be eligible. All haematological malignancies listed in the International Classification of Diseases for Oncology, 3rd Edition^15^ (ICD-O-3) will be eligible.

Review articles, editorials, commentaries and case reports will be excluded along with in vitro, in silico and animal studies. Conference abstracts and other non-peer reviewed publications will also be excluded. Studies that require participants to have a specific infection at the time of study entry (eg, outcomes of patients with a COVID-19 infection where a subgroup had a previous haematological cancer diagnosis) will not be included in the review. To focus on long-term infection outcomes, studies with an average follow-up of less than 1 year from haematological cancer diagnosis in the exposed cohort will be excluded. Studies reporting infections occurring within the first year after diagnosis will not be excluded, provided average overall follow-up extends beyond 1 year. To reduce bias stemming from healthcare-associated exposures and outcomes that are not representative of the general survivor population, studies in which the entire population are hospitalised for the duration of follow-up will also be excluded.

There will be no restriction on the age of study participants to account for the bimodal age distribution of cancers such as acute lymphoblastic leukaemia and Hodgkin’s lymphoma.^16^ All types of infection will be eligible for inclusion in the review, provided there is sufficient evidence that the infection was incident after cancer diagnosis. This is most likely to apply to chronic infections that are associated with an increased risk of developing haematological cancers, such as Epstein–Barr virus,^17 18^ human T-cell lymphotropic virus type-1^18^,^21^ and HIV.^22 23^

### Information sources and search strategy

The two largest biomedical literature databases, MEDLINE and Embase, will be queried using the Ovid interface. In both databases, the search expressions include terms related to (1) haematological malignancies (exposure of interest), (2) infections (outcome) and (3) observational research (eg, cohort studies), combined using Boolean operator terms. The search expressions include terms for Medical Subject Headings (MeSH) and Emtree, as well as key text words with truncation to allow for variations in terminology. A manual search of the reference lists of included papers (backwards citation tracking) will also be completed for any further relevant papers which may have been missed. There will be no search limitations regarding the year of publication and geography. However, only studies published in English will be included in the review to avoid potential performance issues with the NLP algorithm (described below) which will have been primarily trained on English scientific literature. The MEDLINE and Embase search expressions are provided in online supplemental file 2.

### Data management

All retrieved records will be imported into Endnote 21 (Clarivate, Philadelphia, PA, USA). Duplicate entries in MEDLINE and Embase will be detected by the Endnote software and excluded. Additionally, a backup of the search strategy, retrieved records from each database and details of the search date and last update will be securely saved.

### Study selection: general approach and comparison of traditional vs. artificial intelligence supported methods

The records yielded by the database search will be screened for inclusion by systematically applying the inclusion and exclusion criteria in two steps: (1) first, by considering only the information available in the title and abstract; and (2) considering all available information.

In the first step, to aid in the title/abstract screening of studies for this systematic review, we will use ASReview,^13 24^ an open-source tool that uses NLP to analyse study abstracts and predict their relevance based on previous decisions made during the screening process. The abstracts are then ordered from most to least relevant after each screening decision. Once a prespecified number of abstracts have been consecutively screened without inclusion, it can be deduced that no further abstracts will be included and screening can stop.

To assess the reliability of the approach within this review, an internal validation step will compare the NLP-assisted method to a fully manual method on a subset of articles. The internal validation process is described by the following steps:

1. De-duplication of search results using Endnote (as mentioned above).
2. A random sample of 1000 abstracts will be selected and manually screened by two independent reviewers.
  - Discrepancies will be resolved by discussion or with the involvement of a third reviewer.
3. The same 1000 abstracts will be screened separately using ASReview:
  - The tool requires at least one included abstract and one excluded abstract to be provided as ‘prior knowledge’. The ‘prior knowledge’ articles for inclusion will be Carreira *et al*^10^ and Chehab *et al*,^25^ and for exclusion will be Laulund *et al*^26^ and Lee *et al*.^27^
  - Once 200 consecutive abstracts have been screened without inclusion (or all 1000 abstracts are screened if this occurs first), screening will stop.
4. The two approaches will be compared to see if the same articles are selected for full-text screening. The NLP-assisted approach will be considered validated if the manual approach identifies the same articles for full-text screening as the NLP-assisted approach.

Provided the NLP-assisted approach is successfully validated, ASReview will be utilised for title and abstract screening of the remaining articles using the following steps:

1. ‘Prior knowledge’ for ASReview will be all 1000 articles previously screened during the validation step.
2. Once 200 consecutive abstracts have been screened without inclusion, abstract screening will stop.
3. Of the remaining articles that have not been screened, a random sample of 1000 will be taken and manually screened. If any of these articles are eligible for inclusion, the process will be repeated from step 2.
4. Full-text screening of articles will be completed manually.

If the ASReview method fails the validation step, then other NLP-assisted methods will be assessed using a similar approach or a fully manual review may be considered.

### Data items

Data extraction will be performed on all articles included in the review after full-text screening using a pre-defined, standardised data extraction form. This form will be piloted on 3–5 studies to ensure clarity and consistency before being applied to the remaining studies. Data will be extracted by the lead author and independently checked by a co-author, with discrepancies resolved by discussion. Table 1 describes the variables for which data will be sought.

**Table 1:** Characteristics to be extracted from included studies

Relevance	Data needed	Expected format
Study	First author	Forename and surname
	Year of publication	As reported
	Study design	Description of the type of observational study
	Study period	Start and end dates of study
	Length of follow-up	Median (IQR), mean (SD), range
Population	Number of participants	Split by exposure/control
	Geographical location	Setting where the study was conducted
	Age	Median (IQR), mean (SD), range
	Ethnicity	As reported
Exposure	Type(s) of haematological cancer(s) included	As defined in the original studies
	Treatments received since diagnosis	Names of treatments and number of lines of therapies
	Time since diagnosis	Median (IQR), mean (SD), range
Comparator	Definition of control population	Either individuals without cancer or the general population
Outcomes	Type(s) of infection(s) included	As defined in the original studies
	How infection has been defined	eg, positive laboratory test, requirement of antibiotics, antimicrobial resistant infection, hospitalisation, infection-related death
	Quantitative data on infection incidence/mortality for exposed and non-cancer comparison groups	Incidence rate ratios, risk ratios, ORs, HRs or absolute numbers/proportions in the separate exposed and control cohorts

Details on the types of haematological cancers included in the study will be collected along with any available details on the treatments received and the time since diagnosis. The control population will either be defined as individuals with no history of cancer at study entry or the general population. Any methods employed for accounting for vaccination rates (which may be higher in haematological cancer survivors) will also be described.

### Outcomes

The primary outcomes are infection incidence and infection mortality rate. How each study defines infection incidence (eg, positive laboratory test, clinical diagnosis, requirement of antibiotics, hospitalisation) will also be described. All measures of effect that are used to compare the primary outcomes between the exposed and control populations, such as incidence rate ratios, ORs and HRs, will be accepted, as will absolute numbers for both populations. Where available, outcomes will be reported separately in cases where multiple different infection types or haematological cancer types are included in the same study. In addition, whether studies adjust for potential confounders and which confounding variables are adjusted for will be documented.

### Risk-of-bias (quality) assessment

We will use the Newcastle–Ottawa Scale^28^ to assess the risk of bias for each study design. The scale comprises eight items split into three dimensions: selection, comparability and either outcome (cohort studies) or exposure (case–control studies). A point system is used such that studies of higher quality achieve higher scores, up to a maximum of nine.

### Data synthesis

The systematic review will be reported according to the PRISMA statement.^29^ Study characteristics and results will be summarised using tables and descriptive text and graphs, stratified by outcome and potential sources of heterogeneity (e.g. study design, population type). Studies will be grouped based on duration of follow-up for long-term infection outcomes (short: <5 years, medium: 5–10 years, long: 10+ years) as well as by age (<18, ≥18), types of infection described (bacterial, viral, fungal, parasitic, combination) and types of haematological cancer included. An attempt will also be made to differentiate between carrier status and clinical infection.

If at least three of the studies included in the review are sufficiently homogenous in terms of the infection types, outcome definitions and cohort classifications, then we will conduct a meta-analysis using the method of DerSimonian and Laird.^30^ If different effect measures are used across studies that are otherwise appropriate for meta-analysis, we will use appropriate conversions if possible. Higgins and Thompson’s *I*^2^ statistic will be used to quantify heterogeneity. Results of meta-analyses will be illustrated using forest plots. Potential biases in the results of the meta-analyses will be examined using funnel plots and Egger’s regression asymmetry test^31^ if at least 10 studies are included in the analysis.^32^

### Patient and public involvement

Patients and the public were not involved in the design of this systematic review protocol.

## Ethics and dissemination

Ethical approval for this study is not required as no original data will be collected. The results of the review will be disseminated to clinical audiences through conference presentation and submitted to a peer-reviewed journal.

## Supplementary material

10.1136/bmjopen-2025-114803 online supplemental file 1

10.1136/bmjopen-2025-114803 online supplemental file 2
