# Supplementary material for: Long-term infection risks in haematological cancer survivors compared with individuals with no cancer history: protocol for a systematic review aided by artificial intelligence-based methods
Source: BMJ Open. 2026 Mar 18;16(3):e114803. doi: 10.1136/bmjopen-2025-114803 (PMC13007171; doi:10.1136/bmjopen-2025-114803)
Supplement: online supplemental file 2 [file bmjopen-16-3-s002.docx]

**Search terms for EMBASE:**

| # | Searches |
| --- | --- |
| 1 | exp hematologic malignancy/ or exp bone marrow cancer/ or exp myelodysplastic syndrome/ or exp blastic plasmacytoid dendritic cell neoplasm/ or exp bone marrow tumor/ or exp leukemia/ or exp mixed myelodysplastic myeloproliferative disease/ or exp myelofibrosis/ or exp myeloid metaplasia/ or exp myeloma/ or exp myeloproliferative neoplasm/ or exp polycythemia vera/ |
| 2 | (lymphom* or myelom* or myelodysplas* or leuk?emia* or hodgkin* or nonhodgkin* or non-hodgkin* or macroglobulin?emia or (polycyth?emia adj2 vera) or (essential adj2 thrombocyth?emia) or myelofibrosis or myeloproliferative disorder or lymphoproliferative disorder or lymphogranulomato* or reticulosarcom* or burkitt* or lymphosarcom* or (brill adj symmers) or sezary or mycosis fungoides or blood cancer* or (h?ematolog* adj3 malignancy) or (h?ematolog* adj3 cancer) or (h?ematolog* adj3 neoplas*)).mp. |
| 3 | 1 or 2 |
| 4 | exp Infection/ or exp disease transmission/ or exp drug resistant infection/ |
| 5 | (infect* or reactivat* or antimicrob* or AMR or virus or viral or bacteri* or fungus or fungal or sepsis or septic shock or abscess or covid* or influenz* or seropositiv* or seropreval* or communicable or (opportunist* adj2 (infect* or microorganism* or micro-organism* or microb* or pathogen*)) or (MRSA or methicillin-resistan*) or (acinetobacter* or brucellos* or clostridium or enterobacter* or enterococc* or escherichi* or h?emophilus or klebsiell* or legionell* or mycobact* or pneumococc* or proteus or pseudomon* or salmonell* or staphylococc* or stenotrophomon* or streptococc*) or (legionnair* or pneumoni* or pneumocystis) or (adenovir* or coronavir* or enterovir* or parvovir* or rhinovir* or rotavir* or varicella-zoster or VZV) or (respiratory syncytial virus or influenza or parainfluenza) or (cytomegalovir* or cytomegalo-vir*) or (herpes* or shingles) or (hepatitides or hepatitis) or (Measles or Mumps or Rubella) or (aspergill* or candida or candidiais or fusarium or mucorales or entomophthorales or mycosis or mucormycosis or scedosporium) or (zoonoses or zoonotic) or (tuberculos* or koch* disease)).mp. |
| 6 | 4 or 5 |
| 7 | (prevalence* or frequenc* or incidence or risk or risks or rate or rates or ratio or ratios or odds or epidemiolog* or percent* or outcomes or hazard* or cohort).mp. |
| 8 | 3 and 6 and 7 |
| 9 | comment/ or editorial/ or patient education handout/ or "retraction of publication"/ or case reports/ |
| 10 | (editorial or comment or review).pt. |
| 11 | ("systematic review" or "meta-analysis" or "case report").mp. |
| 12 | 9 or 10 or 11 |
| 13 | 8 not 12 |
| 14 | exp Animal/ |
| 15 | exp Humans/ |
| 16 | 14 not 15 |
| 17 | (bovine or murine or mouse or mice or primate or cats or dogs).mp. |
| 18 | exp Veterinary Medicine/ or exp nonhuman/ |
| 19 | 16 or 17 or 18 |
| 20 | 13 not 19 |
| 21 | limit 20 to (english language and "remove preprint records") |

**Search terms for MEDLINE:**

| # | Searches |
| --- | --- |
| 1 | exp hematologic neoplasms/ or exp bone marrow neoplasms/ or exp Myelodysplastic-Myeloproliferative Diseases/ or exp Myelodysplastic syndromes/ or Myeloproliferative Disorders/ or exp leukemia/ or exp lymphoma/ or plasmacytoma/ or waldenstrom macroglobulinemia/ or exp Multiple Myeloma/ |
| 2 | (lymphom* or myelom* or myeloid or myelodysplas* or leuk?emia* or hodgkin* or nonhodgkin* or non-hodgkin* or macroglobulin?emia or (polycyth?emia adj2 vera) or (essential adj2 thrombocyth?emia) or myelofibrosis or myeloproliferative or lymphoproliferative disorder or lymphogranulomato* or histiocy* or reticulosis or reticulosarcom* or burkitt* or lymphosarcom* or bryll-symmer* or sezary or mycosis fungoides or aplast* an?emi* or blood cancer* or (h?ematolog* adj3 malignancy) or (h?ematolog* adj3 cancer) or (h?ematolog* adj3 neoplas*)).mp. |
| 3 | 1 or 2 |
| 4 | exp Infections/ |
| 5 | (infect* or reactivat* or antimicrob* or AMR or virus or viral or bacteri* or fungus or fungal or sepsis or septic shock or abscess or covid* or influenz* or tuberculos* or seropositiv* or seropreval*or communicable or (opportunist* adj2 (infect* or microorganism* or micro-organism* or microb* or pathogen*)) or ((pathogen* or drug resistan*) adj2 (organism* or microorganism* or micro-organism*)) or (MRSA or methicillin-resistan*) or (acinetobacter* or brucellos* or clostridium or enterobacter* or enterococc* or escherichi* or h?emophilus or klebsiell* or legionell* or mycobact* or pneumococc* or proteus or pseudomon* or salmonell* or staphylococc* or stenotrophomon* or streptococc*) or (legionnair* or pneumoni* or pneumocystis) or (adenovir* or coronavir* or enterovir* or parvovir* or rhinovir* or rotavir* or varicella-zoster or VZV) or (respiratory syncytial virus or influenza or parainfluenza) or (cytomegalovir* or cytomegalo-vir*) or (herpes* or shingles) or (hepatitides or hepatitis) or (Measles or Mumps or Rubella) or (aspergill* or candida or candidiais or fusarium or mucorales or entomophthorales or mycosis or mucormycosis or scedosporium) or (zoonoses or zoonotic) or (tuberculos* or koch* disease)).mp. |
| 6 | 4 or 5 |
| 7 | (prevalence* or frequenc* or incidence or risk or risks or rate or rates or ratio or ratios or odds or epidemiolog* or percent* or outcomes or hazard* or cohort).mp. |
| 8 | 3 and 6 and 7 |
| 9 | comment/ or editorial/ or patient education handout/ or "retraction of publication"/ or case reports/ |
| 10 | (editorial or comment or review).pt. |
| 11 | ("systematic review" or "meta-analysis" or "case report").mp. |
| 12 | 9 or 10 or 11 |
| 13 | 8 not 12 |
| 14 | exp Animals/ |
| 15 | exp Humans/ |
| 16 | 14 not 15 |
| 17 | (bovine or murine or mouse or mice or primate or cats or dogs).mp. |
| 18 | exp Veterinary Medicine/ |
| 19 | 16 or 17 or 18 |
| 20 | 13 not 19 |
| 21 | limit 20 to (english language and "remove preprint records") |
