# Supplementary material for: Long-term infection risks in haematological cancer survivors compared with individuals with no cancer history: protocol for a systematic review aided by artificial intelligence-based methods
Source: BMJ Open. 2026 Mar 18;16(3):e114803. doi: 10.1136/bmjopen-2025-114803 (PMC13007171; doi:10.1136/bmjopen-2025-114803)
Supplement: Supplementary file 3 [file bmjopen-2025-114803.reviewer_comments.pdf]

PEER REVIEW HISTORY

BMJ Open publishes all reviews undertaken for accepted manuscripts. Reviewers are asked to complete a checklist review form and are provided with free text boxes to elaborate on their assessment. These free text comments are reproduced below.

ARTICLE DETAILS

Title (Provisional)

Long-term infection risks in haematological cancer survivors compared with individuals with no cancer history: protocol for a systematic review aided by artificial intelligence-based methods

Authors

Wilson, William; Forbes, Harriet; Hazell, Matthew; Hopkins, Lily; Funston, Garth; O'Reilly, Maeve; Bhaskaran, Krishnan; Carreira, Helena

---

VERSION 1 - REVIEW

**Reviewer**	**1**
**Name**	**Smith, Alexandra**
**Affiliation**	**University Of York, Health Sciences**
**Date**	**07-Feb-2026**
**COI**	**None**

This is a well written manuscript. The methodology described is standard, but with a novel aspect of utilising AI to ameliorate the challenging issue of conducting a review around long-term infection risk in survivors of haematological cancer due to the extensive literature. Reassuringly, the authors propose to undertake a validation step comparing 1000 abstracts manually screened compared to those screened using ASReviewer.

My only major concern is that the review proposes to only examine infections from the year after diagnosis as the authors state that it is hoping to capture effects after the initial treatment period. Whilst this will be true for haematological cancers that are potentially curable such as acute myeloid leukaemia, diffuse large B cell lymphoma and Hodgkin lymphoma a significant proportion of haematological cancers are now perceived as chronic conditions; including myeloma, chronic lymphocytic leukaemia and follicular lymphoma. These conditions may either have periods of active monitoring (watch and wait) or are on long-term therapy depending on their relapsing-remitting status.

VERSION 1 - AUTHOR RESPONSE

Reviewer: 1

Comments to the Author:
This is a well written manuscript. The methodology described is standard, but with a novel aspect of utilising AI to ameliorate the challenging issue of conducting a review around long-term infection risk in survivors of haematological cancer due to the extensive literature. Reassuringly, the authors propose to undertake a validation step comparing 1000 abstracts manually screened compared to those screened using ASReviewer.

My only major concern is that the review proposes to only examine infections from the year after diagnosis as the authors state that it is hoping to capture effects after the initial treatment period. Whilst this will be true for haematological cancers that are potentially curable such as acute myeloid leukaemia, diffuse large B cell lymphoma and Hodgkin lymphoma a significant proportion of haematological cancers are now perceived as chronic conditions; including myeloma, chronic lymphocytic leukaemia and follicular lymphoma. These conditions may either have periods of active monitoring (watch and wait) or are on long-term therapy depending on their relapsing-remitting status.

Response:
Thank you for taking the time to review our manuscript and provide thoughtful and constructive comments. We are grateful for the positive feedback regarding the clarity of the manuscript and the novelty of incorporating AI-assisted screening with an appropriate planned validation step.

We appreciate the concern raised regarding the definition of the post-diagnosis period and acknowledge our wording was unclear. To clarify, the review does not exclude studies examining infections within the first year after diagnosis, provided that the average follow-up extends beyond one year. One of the primary aims of the review is to assess whether infection risk varies over time following diagnosis; studies with less than one year of follow-up would not allow meaningful evaluation of longer-term risk. We anticipate that the review may identify differences in infection risk over time across haematological cancer subtypes, potentially reflecting the differing treatment strategies and disease trajectories you highlight.

We have revised the Methods section to clarify this point. We have also removed the previous wording in the Discussion suggesting that the review focused only on infection risk at least one year after diagnosis, as this was misleading.

Editor's comments:

Please ensure that your abstract is formatted according to our Instructions for Authors: https://bmjopen.bmj.com/pages/authors/#protocol.

This has been done.

Please include the planned start and end dates for the study in the methods section. Please also state and explain any discrepancies between the timeline in the PROSPERO record and the manuscript.

This has been added (and PROSPERO planned end date updated).

The discussion section is optional for protocols and if present we would like it to focus on discussing the study's strengths and weaknesses. Please consider removing it. Alternatively, please revise appropriately.

The discussion has been removed.

Please ensure that the main text contains an ethics and dissemination section as per our instructions for authors: https://bmjopen.bmj.com/pages/authors/#protocol

This has been added (lines 224-227).

You indicated in the PRISMA-P checklist that you are planning to assess the certainty of evidence. We cannot see this plan within the manuscript. Please either add details to the manuscript or revise the checklist.

PRISMA-P checklist has been revised.
